# 2,2′-{[2-(Pyridin-2-yl)-1,3-diazinane-1,3-diyl]bis(methylene)}diphenol

**DOI:** 10.1107/S1600536812035477

**Published:** 2012-08-23

**Authors:** Adailton J. Bortoluzzi, Geovana G. Terra

**Affiliations:** a Depto. de Química, Universidade Federal de Santa Catarina, 88040-900 - Florianópolis, SC, Brazil

## Abstract

The title compound, C_23_H_25_N_3_O_2_, was obtained as an inter­mediary in the preparation of non-symmetric tertiary diamines. The mol­ecular structure presents T-shaped spatial form, in which the pyrimidine ring exhibits a chair conformation. The pyridyl ring is almost perpendicular to the phenyl rings with dihedral angles of 80.17 (8) and 76.03 (2)°. The phenol and amine groups are involved in two strong intra­molecular O—H⋯N inter­actions. In the crystal, the mol­ecules are stacked along [010]; however, no inter­molecular inter­actions are observed.

## Related literature

For the synthetic procedure, see: Hureau *et al.* (2008 ▶). For related structures, see: Yokoyama *et al.* (1995 ▶); Xia *et al.* (2007 ▶). For standard bond lengths and angles, see: Bruno *et al.* (2004 ▶).
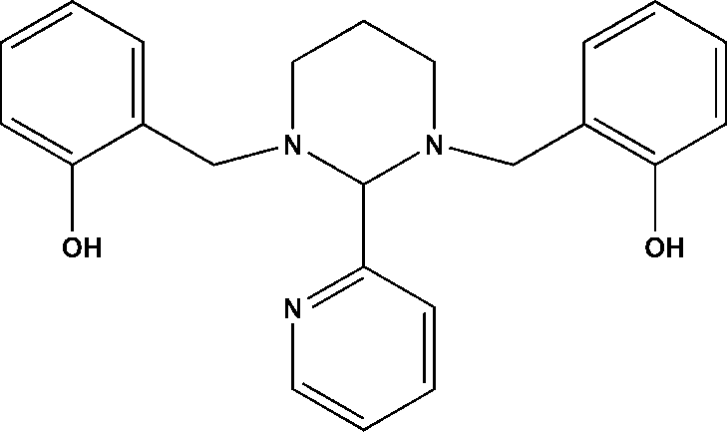


## Experimental

### 

#### Crystal data

C_23_H_25_N_3_O_2_
*M* *_r_* = 375.46
Monoclinic, 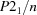

*a* = 18.7615 (16) Å
*b* = 6.2105 (11) Å
*c* = 19.0407 (12) Å
β = 114.594 (8)°
*V* = 2017.3 (4) Å^3^
*Z* = 4
Mo *K*α radiation
μ = 0.08 mm^−1^
*T* = 293 K
0.50 × 0.50 × 0.40 mm

#### Data collection

Enraf–Nonius CAD-4 diffractometer
3711 measured reflections
3595 independent reflections
2038 reflections with *I* > 2σ(*I*)
*R*_int_ = 0.079
3 standard reflections every 200 reflections intensity decay: 1%

#### Refinement

*R*[*F* ^2^ > 2σ(*F* ^2^)] = 0.052
*wR*(*F* ^2^) = 0.146
*S* = 1.01
3595 reflections
253 parameters
H-atom parameters constrained
Δρ_max_ = 0.17 e Å^−3^
Δρ_min_ = −0.22 e Å^−3^

### 

Data collection: *CAD-4 Software* (Enraf–Nonius, 1989 ▶); cell refinement: *SET4* in *CAD-4 Software*; data reduction: *HELENA* (Spek, 1996 ▶); program(s) used to solve structure: *SIR97* (Altomare *et al.*, 1999 ▶); program(s) used to refine structure: *SHELXL97* (Sheldrick, 2008 ▶); molecular graphics: *PLATON* (Spek, 2009 ▶); software used to prepare material for publication: *SHELXL97*.

## Supplementary Material

Crystal structure: contains datablock(s) global, I. DOI: 10.1107/S1600536812035477/lr2078sup1.cif

Structure factors: contains datablock(s) I. DOI: 10.1107/S1600536812035477/lr2078Isup2.hkl

Supplementary material file. DOI: 10.1107/S1600536812035477/lr2078Isup3.mol

Supplementary material file. DOI: 10.1107/S1600536812035477/lr2078Isup4.cml

Additional supplementary materials:  crystallographic information; 3D view; checkCIF report

## Figures and Tables

**Table 1:** Hydrogen-bond geometry (Å, °)

*D*—H⋯*A*	*D*—H	H⋯*A*	*D*⋯*A*	*D*—H⋯*A*
O10—H10⋯N1	1.02	1.69	2.624 (3)	150
O20—H20⋯N5	1.09	1.72	2.705 (3)	148

**Fig. 1.**
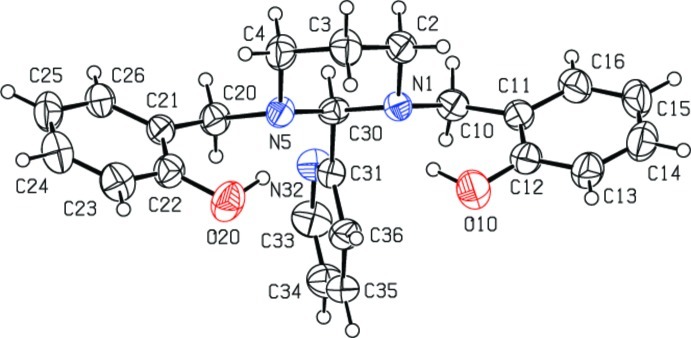
The molecular structure of the title compound. Ellipsoids are shown at the 40% probability level.

## supplementary crystallographic information

### Comment

The molecular structure of the title compound (I) shows T-shaped spatial form (Fig. 1). Pyrimidine ring adopts regular chair conformation with square plane formed by N1/C2/C4/C5 atoms (r.m.s. deviation = 0.0111).

The dihedral angles between the mean planes of the rings C31/C36 and C11/C16 of 80.17 (8)° and C31/36 and C211/C26 of 76.03 (8)° demonstrate that pyridil ring is almost perpendicular to phenol groups.

Two strong intramolecular O—H···N hydrogen bonds between phenol and amine groups (Table 1) form additional six-membered rings, which contribute for the rigidity of the structure and avoid the crystal supramolecurity.

The molecules are stacked along [010] direction, however no further intermolecular interactions, such as π-stacking, were observed.

All bond lengths and angles found for (I) are in the expected range for organic compounds (Bruno *et al.*, 2004).

### Experimental

Compound (I) was synthesized according to the procedure described by Hureau *et al.* (2008).

A solution containing 6.0 g of *N,N'*-*bis*(2-hydroxybenzyl)-1,3-diamino-propane (21,3 mmol) and 2.39 g (21,3 mmol) of 2-pyridinecarboxaldehyde in 60 ml of MeOH was stirred at temperature of 333,15 K for 1 h. The solvent was evaporated under reduced pressure to afford a white precipitate, which was filtered off and washed with dry diethyl eter. (85% yield = 85%). MP 154.6–154.9 °C, EA for C_23_H_25_N_3_O_2_: calc C 73,53%; H 7,18%; N 11,27%, found C, 73.57%; H, 6.71%; N, 11.195.

### Refinement

H atoms attached to carbon atoms were placed at their idealized positions with distances of 0.98 and 0.97 Å and *U*_iso_ fixed at 1.2 times of *U*_eq_ of the preceding atom for CH and CH_2_, respectively. H atoms of the hydroxyl groups were found from difference map and treated with riding model and their *U*_iso_ were fixed at 1.2 times of *U*_eq_ of the parent atom.

### Crystal data

**Table:**

C_23_H_25_N_3_O_2_	*F*(000) = 800
*M**_r_* = 375.46	*D*_x_ = 1.236 Mg m^−^^3^
Monoclinic, *P*2_1_/*n*	Mo *K*α radiation, λ = 0.71073 Å
Hall symbol: -P 2yn	Cell parameters from 25 reflections
*a* = 18.7615 (16) Å	θ = 5.5–17.4°
*b* = 6.2105 (11) Å	µ = 0.08 mm^−^^1^
*c* = 19.0407 (12) Å	*T* = 293 K
β = 114.594 (8)°	Block, colorless
*V* = 2017.3 (4) Å^3^	0.50 × 0.50 × 0.40 mm
*Z* = 4	

### Data collection

**Table:**

Enraf–Nonius CAD-4 diffractometer	*R*_int_ = 0.079
Radiation source: fine-focus sealed tube	θ_max_ = 25.1°, θ_min_ = 1.3°
Graphite monochromator	*h* = −20→22
ω–2θ scans	*k* = −7→0
3711 measured reflections	*l* = −22→0
3595 independent reflections	3 standard reflections every 200 reflections
2038 reflections with *I* > 2σ(*I*)	intensity decay: 1%

### Refinement

**Table:**

Refinement on *F*^2^	Primary atom site location: structure-invariant direct methods
Least-squares matrix: full	Secondary atom site location: difference Fourier map
*R*[*F*^2^ > 2σ(*F*^2^)] = 0.052	Hydrogen site location: inferred from neighbouring sites
*wR*(*F*^2^) = 0.146	H-atom parameters constrained
*S* = 1.01	*w* = 1/[σ^2^(*F*_o_^2^) + (0.0711*P*)^2^ + 0.2051*P*] where *P* = (*F*_o_^2^ + 2*F*_c_^2^)/3
3595 reflections	(Δ/σ)_max_ < 0.001
253 parameters	Δρ_max_ = 0.17 e Å^−^^3^
0 restraints	Δρ_min_ = −0.22 e Å^−^^3^

### Fractional atomic coordinates and isotropic or equivalent isotropic displacement parameters (Å^2^)

**Table:**

	*x*	*y*	*z*	*U*_iso_*/*U*_eq_	
N1	0.65802 (10)	0.2353 (3)	−0.10455 (10)	0.0445 (5)	
C2	0.57867 (13)	0.1394 (4)	−0.14032 (13)	0.0545 (7)	
H2A	0.5828	−0.0130	−0.1492	0.065*	
H2B	0.5488	0.2073	−0.1898	0.065*	
C3	0.53699 (14)	0.1696 (5)	−0.08857 (14)	0.0636 (8)	
H3A	0.5285	0.3218	−0.0835	0.076*	
H3B	0.4863	0.0989	−0.1109	0.076*	
C4	0.58552 (14)	0.0754 (5)	−0.01042 (14)	0.0631 (8)	
H4A	0.5594	0.0982	0.0235	0.076*	
H4B	0.5914	−0.0784	−0.0151	0.076*	
N5	0.66299 (10)	0.1781 (3)	0.02244 (11)	0.0481 (5)	
C10	0.69848 (14)	0.2035 (4)	−0.15583 (14)	0.0528 (6)	
H10A	0.6964	0.0521	−0.1691	0.063*	
H10B	0.7532	0.2433	−0.1283	0.063*	
C11	0.66255 (14)	0.3345 (4)	−0.22904 (13)	0.0504 (6)	
C12	0.63385 (15)	0.5401 (4)	−0.22823 (15)	0.0581 (7)	
C13	0.60453 (16)	0.6654 (5)	−0.29458 (16)	0.0697 (8)	
H13	0.5838	0.8009	−0.2938	0.084*	
C14	0.60633 (17)	0.5889 (6)	−0.36109 (17)	0.0781 (9)	
H14	0.5881	0.6747	−0.4051	0.094*	
C15	0.63473 (17)	0.3875 (6)	−0.36363 (16)	0.0741 (9)	
H15	0.6353	0.3361	−0.4093	0.089*	
C16	0.66272 (15)	0.2605 (5)	−0.29765 (15)	0.0635 (7)	
H16	0.6819	0.1235	−0.2995	0.076*	
C20	0.70860 (14)	0.0956 (4)	0.10175 (13)	0.0536 (7)	
H20A	0.7614	0.1532	0.1214	0.064*	
H20B	0.7121	−0.0600	0.1000	0.064*	
C21	0.67202 (13)	0.1560 (4)	0.15583 (13)	0.0479 (6)	
C22	0.64091 (15)	0.3595 (5)	0.15448 (14)	0.0564 (7)	
C23	0.61059 (16)	0.4140 (5)	0.20724 (16)	0.0689 (8)	
H23	0.5892	0.5500	0.2057	0.083*	
C24	0.61217 (17)	0.2666 (6)	0.26183 (16)	0.0735 (8)	
H24	0.5921	0.3038	0.2974	0.088*	
C25	0.64312 (17)	0.0652 (5)	0.26416 (15)	0.0703 (8)	
H25	0.6443	−0.0341	0.3012	0.084*	
C26	0.67240 (14)	0.0119 (4)	0.21108 (13)	0.0568 (7)	
H26	0.6930	−0.1250	0.2125	0.068*	
C30	0.70474 (12)	0.1435 (4)	−0.02717 (12)	0.0443 (6)	
H30	0.7115	−0.0114	−0.0321	0.053*	
C31	0.78452 (13)	0.2502 (4)	0.00853 (13)	0.0451 (6)	
N32	0.84615 (11)	0.1185 (3)	0.03432 (12)	0.0549 (6)	
C33	0.91701 (15)	0.2096 (5)	0.06679 (17)	0.0691 (8)	
H33	0.9605	0.1195	0.0861	0.083*	
C34	0.92949 (17)	0.4257 (6)	0.07337 (17)	0.0740 (9)	
H34	0.9801	0.4810	0.0955	0.089*	
C35	0.86581 (17)	0.5606 (5)	0.04665 (14)	0.0638 (8)	
H35	0.8723	0.7092	0.0505	0.077*	
C36	0.79262 (15)	0.4714 (4)	0.01433 (14)	0.0550 (7)	
H36	0.7485	0.5592	−0.0037	0.066*	
O10	0.63242 (12)	0.6251 (3)	−0.16299 (11)	0.0789 (6)	
H10	0.6485	0.5027	−0.1240	0.095*	
O20	0.63844 (12)	0.5107 (3)	0.10137 (11)	0.0754 (6)	
H20	0.6531	0.4237	0.0597	0.090*	

### Atomic displacement parameters (Å^2^)

**Table:**

	*U*^11^	*U*^22^	*U*^33^	*U*^12^	*U*^13^	*U*^23^
N1	0.0433 (11)	0.0465 (11)	0.0424 (10)	0.0008 (9)	0.0167 (9)	0.0002 (9)
C2	0.0463 (14)	0.0617 (17)	0.0479 (13)	−0.0021 (13)	0.0120 (11)	0.0025 (13)
C3	0.0396 (13)	0.093 (2)	0.0531 (15)	−0.0052 (14)	0.0141 (12)	0.0054 (15)
C4	0.0490 (15)	0.084 (2)	0.0529 (15)	−0.0118 (14)	0.0176 (12)	0.0097 (15)
N5	0.0394 (11)	0.0558 (13)	0.0452 (11)	−0.0027 (10)	0.0136 (9)	0.0079 (10)
C10	0.0543 (15)	0.0494 (15)	0.0565 (15)	0.0046 (12)	0.0249 (13)	−0.0022 (12)
C11	0.0479 (14)	0.0565 (16)	0.0476 (14)	−0.0053 (12)	0.0206 (11)	−0.0021 (13)
C12	0.0621 (17)	0.0532 (16)	0.0529 (16)	−0.0021 (14)	0.0178 (13)	0.0038 (14)
C13	0.0732 (19)	0.0683 (19)	0.0600 (17)	−0.0021 (16)	0.0202 (15)	0.0139 (16)
C14	0.069 (2)	0.100 (3)	0.0585 (19)	−0.0141 (19)	0.0190 (15)	0.0212 (19)
C15	0.0710 (19)	0.108 (3)	0.0508 (17)	−0.022 (2)	0.0330 (15)	−0.0036 (18)
C16	0.0602 (16)	0.078 (2)	0.0611 (17)	−0.0102 (15)	0.0337 (14)	−0.0119 (16)
C20	0.0468 (14)	0.0567 (16)	0.0479 (14)	0.0035 (12)	0.0104 (11)	0.0101 (13)
C21	0.0412 (13)	0.0541 (15)	0.0392 (13)	−0.0039 (12)	0.0076 (10)	0.0041 (12)
C22	0.0530 (15)	0.0593 (17)	0.0517 (15)	0.0035 (14)	0.0167 (12)	0.0081 (14)
C23	0.0653 (18)	0.073 (2)	0.0637 (17)	0.0132 (16)	0.0224 (15)	0.0025 (16)
C24	0.074 (2)	0.094 (2)	0.0539 (17)	0.0093 (19)	0.0283 (15)	0.0021 (18)
C25	0.0755 (19)	0.085 (2)	0.0472 (15)	0.0029 (18)	0.0225 (14)	0.0134 (16)
C26	0.0556 (15)	0.0609 (17)	0.0450 (14)	0.0030 (13)	0.0121 (12)	0.0065 (13)
C30	0.0408 (13)	0.0365 (12)	0.0505 (14)	0.0009 (11)	0.0140 (11)	0.0029 (11)
C31	0.0461 (14)	0.0459 (15)	0.0437 (13)	0.0016 (12)	0.0191 (11)	0.0029 (12)
N32	0.0421 (12)	0.0536 (13)	0.0643 (13)	0.0055 (11)	0.0176 (10)	0.0002 (11)
C33	0.0424 (16)	0.077 (2)	0.078 (2)	0.0063 (15)	0.0156 (14)	−0.0024 (17)
C34	0.0540 (17)	0.093 (3)	0.0694 (19)	−0.0226 (18)	0.0199 (15)	−0.0098 (18)
C35	0.076 (2)	0.0546 (17)	0.0557 (16)	−0.0227 (16)	0.0219 (15)	−0.0059 (14)
C36	0.0592 (17)	0.0457 (16)	0.0567 (15)	0.0011 (13)	0.0207 (13)	0.0039 (13)
O10	0.1205 (17)	0.0512 (12)	0.0618 (12)	0.0213 (12)	0.0346 (12)	0.0043 (10)
O20	0.0978 (15)	0.0595 (12)	0.0769 (13)	0.0172 (11)	0.0444 (11)	0.0199 (11)

### Geometric parameters (Å, º)

**Table:**

N1—C10	1.478 (3)	C20—C21	1.504 (3)
N1—C30	1.480 (3)	C20—H20A	0.9700
N1—C2	1.480 (3)	C20—H20B	0.9700
C2—C3	1.503 (3)	C21—C26	1.379 (3)
C2—H2A	0.9700	C21—C22	1.388 (4)
C2—H2B	0.9700	C22—O20	1.367 (3)
C3—C4	1.503 (3)	C22—C23	1.387 (4)
C3—H3A	0.9700	C23—C24	1.376 (4)
C3—H3B	0.9700	C23—H23	0.9300
C4—N5	1.468 (3)	C24—C25	1.372 (4)
C4—H4A	0.9700	C24—H24	0.9300
C4—H4B	0.9700	C25—C26	1.376 (4)
N5—C30	1.472 (3)	C25—H25	0.9300
N5—C20	1.483 (3)	C26—H26	0.9300
C10—C11	1.509 (3)	C30—C31	1.515 (3)
C10—H10A	0.9700	C30—H30	0.9800
C10—H10B	0.9700	C31—N32	1.332 (3)
C11—C16	1.386 (3)	C31—C36	1.382 (3)
C11—C12	1.388 (4)	N32—C33	1.336 (3)
C12—O10	1.360 (3)	C33—C34	1.359 (4)
C12—C13	1.388 (4)	C33—H33	0.9300
C13—C14	1.366 (4)	C34—C35	1.372 (4)
C13—H13	0.9300	C34—H34	0.9300
C14—C15	1.368 (4)	C35—C36	1.367 (3)
C14—H14	0.9300	C35—H35	0.9300
C15—C16	1.388 (4)	C36—H36	0.9300
C15—H15	0.9300	O10—H10	1.0163
C16—H16	0.9300	O20—H20	1.0857
			
C10—N1—C30	110.60 (17)	N5—C20—C21	112.13 (19)
C10—N1—C2	109.68 (18)	N5—C20—H20A	109.2
C30—N1—C2	111.63 (18)	C21—C20—H20A	109.2
N1—C2—C3	110.3 (2)	N5—C20—H20B	109.2
N1—C2—H2A	109.6	C21—C20—H20B	109.2
C3—C2—H2A	109.6	H20A—C20—H20B	107.9
N1—C2—H2B	109.6	C26—C21—C22	118.3 (2)
C3—C2—H2B	109.6	C26—C21—C20	120.1 (2)
H2A—C2—H2B	108.1	C22—C21—C20	121.5 (2)
C4—C3—C2	109.6 (2)	O20—C22—C23	118.1 (3)
C4—C3—H3A	109.8	O20—C22—C21	121.6 (2)
C2—C3—H3A	109.8	C23—C22—C21	120.3 (3)
C4—C3—H3B	109.8	C24—C23—C22	119.9 (3)
C2—C3—H3B	109.8	C24—C23—H23	120.0
H3A—C3—H3B	108.2	C22—C23—H23	120.0
N5—C4—C3	109.7 (2)	C25—C24—C23	120.4 (3)
N5—C4—H4A	109.7	C25—C24—H24	119.8
C3—C4—H4A	109.7	C23—C24—H24	119.8
N5—C4—H4B	109.7	C24—C25—C26	119.2 (3)
C3—C4—H4B	109.7	C24—C25—H25	120.4
H4A—C4—H4B	108.2	C26—C25—H25	120.4
C4—N5—C30	111.11 (18)	C25—C26—C21	121.8 (3)
C4—N5—C20	109.47 (18)	C25—C26—H26	119.1
C30—N5—C20	111.56 (18)	C21—C26—H26	119.1
N1—C10—C11	112.53 (19)	N5—C30—N1	109.26 (17)
N1—C10—H10A	109.1	N5—C30—C31	109.79 (18)
C11—C10—H10A	109.1	N1—C30—C31	110.09 (18)
N1—C10—H10B	109.1	N5—C30—H30	109.2
C11—C10—H10B	109.1	N1—C30—H30	109.2
H10A—C10—H10B	107.8	C31—C30—H30	109.2
C16—C11—C12	117.9 (2)	N32—C31—C36	122.1 (2)
C16—C11—C10	121.1 (2)	N32—C31—C30	116.2 (2)
C12—C11—C10	120.8 (2)	C36—C31—C30	121.7 (2)
O10—C12—C13	117.6 (3)	C31—N32—C33	117.1 (2)
O10—C12—C11	121.5 (2)	N32—C33—C34	124.1 (3)
C13—C12—C11	120.9 (3)	N32—C33—H33	118.0
C14—C13—C12	119.7 (3)	C34—C33—H33	118.0
C14—C13—H13	120.1	C33—C34—C35	118.6 (3)
C12—C13—H13	120.1	C33—C34—H34	120.7
C13—C14—C15	120.7 (3)	C35—C34—H34	120.7
C13—C14—H14	119.6	C36—C35—C34	118.4 (3)
C15—C14—H14	119.6	C36—C35—H35	120.8
C14—C15—C16	119.6 (3)	C34—C35—H35	120.8
C14—C15—H15	120.2	C35—C36—C31	119.7 (3)
C16—C15—H15	120.2	C35—C36—H36	120.1
C11—C16—C15	121.1 (3)	C31—C36—H36	120.1
C11—C16—H16	119.5	C12—O10—H10	105.3
C15—C16—H16	119.5	C22—O20—H20	104.9
			
C10—N1—C2—C3	179.7 (2)	C20—C21—C22—C23	−177.2 (2)
C30—N1—C2—C3	56.7 (3)	O20—C22—C23—C24	−179.8 (3)
N1—C2—C3—C4	−56.0 (3)	C21—C22—C23—C24	0.8 (4)
C2—C3—C4—N5	57.8 (3)	C22—C23—C24—C25	−0.4 (4)
C3—C4—N5—C30	−60.3 (3)	C23—C24—C25—C26	−0.2 (4)
C3—C4—N5—C20	176.1 (2)	C24—C25—C26—C21	0.5 (4)
C30—N1—C10—C11	−167.62 (19)	C22—C21—C26—C25	−0.1 (4)
C2—N1—C10—C11	68.8 (2)	C20—C21—C26—C25	176.6 (2)
N1—C10—C11—C16	−148.2 (2)	C4—N5—C30—N1	59.5 (2)
N1—C10—C11—C12	36.9 (3)	C20—N5—C30—N1	−178.04 (17)
C16—C11—C12—O10	−179.3 (2)	C4—N5—C30—C31	−179.67 (19)
C10—C11—C12—O10	−4.3 (4)	C20—N5—C30—C31	−57.2 (2)
C16—C11—C12—C13	1.5 (4)	C10—N1—C30—N5	179.92 (18)
C10—C11—C12—C13	176.6 (2)	C2—N1—C30—N5	−57.7 (2)
O10—C12—C13—C14	178.5 (2)	C10—N1—C30—C31	59.3 (2)
C11—C12—C13—C14	−2.3 (4)	C2—N1—C30—C31	−178.30 (19)
C12—C13—C14—C15	1.8 (4)	N5—C30—C31—N32	111.9 (2)
C13—C14—C15—C16	−0.6 (4)	N1—C30—C31—N32	−127.7 (2)
C12—C11—C16—C15	−0.3 (4)	N5—C30—C31—C36	−67.7 (3)
C10—C11—C16—C15	−175.4 (2)	N1—C30—C31—C36	52.7 (3)
C14—C15—C16—C11	−0.1 (4)	C36—C31—N32—C33	0.3 (4)
C4—N5—C20—C21	−64.3 (3)	C30—C31—N32—C33	−179.3 (2)
C30—N5—C20—C21	172.29 (19)	C31—N32—C33—C34	−1.4 (4)
N5—C20—C21—C26	141.8 (2)	N32—C33—C34—C35	1.4 (5)
N5—C20—C21—C22	−41.6 (3)	C33—C34—C35—C36	−0.2 (4)
C26—C21—C22—O20	−179.9 (2)	C34—C35—C36—C31	−0.7 (4)
C20—C21—C22—O20	3.4 (4)	N32—C31—C36—C35	0.7 (4)
C26—C21—C22—C23	−0.5 (4)	C30—C31—C36—C35	−179.7 (2)

### Hydrogen-bond geometry (Å, º)

**Table:**

*D*—H···*A*	*D*—H	H···*A*	*D*···*A*	*D*—H···*A*
O10—H10···N1	1.02	1.69	2.624 (3)	150
O20—H20···N5	1.09	1.72	2.705 (3)	148
